# Effect of Surface Treatment on Flexural and Tribological Properties of Poly(p-phenylene Benzobisoxazole)/Polyimide Composites under Normal and Elevated Temperature

**DOI:** 10.3390/ma11112131

**Published:** 2018-10-30

**Authors:** Liang Yu, Ren He, Yuanjie Zhang, Jicheng Gao

**Affiliations:** Mechanical Engineering College of Yangzhou University, Yangzhou 225127, China; 03131187@cumt.edu.cn (R.H.); 15861394939@163.com (Y.Z.); gaojicheng1984@163.com (J.G.)

**Keywords:** elevated temperature, flexural properties, tribological properties, PBO fiber, surface treatment

## Abstract

(1) In order to improve the interface bonding state between poly(p-phenylene benzobisoxazole) (PBO) fibers and a polyimide (PI) polymer matrix, as well as its effectiveness under elevated temperature, rare earth solution (RES) and coupling agent were employed toward PBO fibers as surface modifiers in this article, respectively. (2) The surface characteristics of the PBO fibers before and after modifications were characterized and analyzed by X-ray photoelectron spectroscopy (XPS) and Fourier transform infrared spectroscopy (FT-IR). The effects of the surface treatment of the PBO fibers on the flexural properties and friction and wear behaviors of the polyimide polymer matrix composites reinforced by PBO fibers (PBO/PI) were studied under normal and elevated temperature, and the morphologies of the bending fracture and the worn surface were observed through scanning electronic microscopy (SEM). (3) The results revealed that the RES modification was superior to the coupling agent modification regarding increasing the surface activity of the PBO fibers. (4) The PBO/PI composite treated by RES had higher flexural properties and more excellent anti-friction and wear resistance than the pretreated and coupling agent-treated composites under both normal and elevated temperatures.

## 1. Introduction

Poly(p-phenylene benzobisoxazole) (PBO) fiber is a kind of high-performance polymer material that exhibits some extraordinary characteristics with unique extended rigid-rod molecular configuration [1], such as excellent thermal stability, higher anti-abrasion, remarkable mechanical property, and dramatic resistance to creep, flame, and chemicals [2,3,4]. In addition, PBO fiber has a smooth molecular structure, low polarity, and low surface energy, resulting in a relatively low coefficient of friction. PBO fiber has superior creep resistance and shear resistance compared with aramid fiber [5], does not exhibit the brittle weakness of carbon fiber and glass fiber, and does not scratch the surface of the counterpart during the rubbing process. The above characteristics make the PBO fiber a particularly excellent anti-friction and wear-resistant material. The heat resistance of PBO is high in comparison with the other organic polymers [6,7]. As an example, PBO fiber degrades at about 600 °C and form a 3 wt % residue at 1200 °C in the air [8]. As a heat-resistant fiber showing excellent mechanical properties at elevated temperature, PBO fiber-reinforced polymer composites possess excellent thermal stability, high specific strength, and superb environmental resistance [9], and have become materials of choice for high-temperature application domains of military installations, aerospace crafts, and common industry [10,11,12].

Although the mechanical properties of short PBO fiber-reinforced polymer matrix composites (SPRP) are weak compared with continuous PBO fiber-reinforced polymer matrix composites (CPRP), SPRP can overcome disadvantages such as high manufacturing cost and a low precision of products. In addition, SPRP can be processed directly using standard metal machining equipment due to good dimensional stability at processing temperatures, and can achieve dimensional tolerances that are difficult to achieve for CPRP [13], which is suitable for preparing rubbing pair materials with complex shape and excellent anti-friction and wear resistance. Therefore, SPRP may have good comprehensive properties, a wide range of processing techniques, and broad application prospects. However, the properties of composites reinforced by short fibers depend strongly on the efficiency of stress transfer at the bonding interface of fiber/matrix, in addition to the properties associated with the various components, especially in a high-temperature environment [14,15,16]. The characteristics of the PBO fiber surface are relatively smooth and chemically inactive, which results in its low surface energy and poor adhesion with the resin matrix [17]. Therefore, several techniques for surface treatments on PBO fibers have been utilized to ameliorate the interface bonding state between polymer matrices and PBO fibers. Tamargo-Martinez et al. modified PBO fiber surfaces using the plasma treatment, and they found that the work produced an increase in the adhesion of PBO fibers to epoxy resin [18]. Zhang et al. employed the methods of copolymerization to introduce active functional groups into the macromolecular chains of PBO. The results indicated that the interfacial shear strength between fibers and matrices increased remarkably [19]. Coupling agent [20], enzymatic catalyst [21], and γ-ray irradiation [22] modification techniques were also proposed. These methods all can effectively improve the surface chemical inertness of PBO fiber. However, the effects of the surface modification of PBO fiber on the flexural properties and friction and wear behaviors of its composites under elevated temperature have been rarely reported in the literature.

Excellent interfacial bonding properties are needed in high-temperature environments for advanced polymer matrix composites in order for them to achieve better load-carrying capacity and wear resistance. Generally, the flexural strength of the composite materials can reflect the bond strength between the polymer matrix and the reinforcing materials [23]. In this work, the modification method of rare earth solution (RES) was used for the PBO fibers to improve the interfacial bonding properties of the composite. As a reference, a relatively mature modification method of the coupling agent was employed as a parallel test. The effects of RES and coupling agent treatment on the flexural properties and friction and wear behaviors of PI composites were evaluated under normal and elevated temperature, respectively.

## 2. Materials and Methods

### 2.1. Materials

In the current study, the PBO fiber that was used as the reinforcement was produced commercially by Toyobo Co. Ltd. (Kita-ku, Osaka, Japan) with the trade name Zylon™ AS. The performance parameters provided by Toyobo Co. Ltd. are as follows: tensile strength, 5800 MPa; tensile modulus, 180 GPa; thermal decomposition temperature, 650 °C; density, 1.54 g/cm^3^; diameter, approximately 12.8 μm; aspect ratio, 234. The polyimide (PI) resin was self-made in Shanghai Research Institute of synthetic resins.

The main component in the RE solution was LaCl_3_, which was purchased from Shanghai Yuelong New Materials Co. Ltd. (Shanghai, China). The main component in the coupling agents solution was 3-aminopropyltriethoxysilane (APTES), which was purchased from Aldrich Chemical Company (Milwaukee, WI, USA). The other chemical reagents used in this work were commercially obtained without further purification.

### 2.2. PBO Fiber Modification

**Figure 1:**
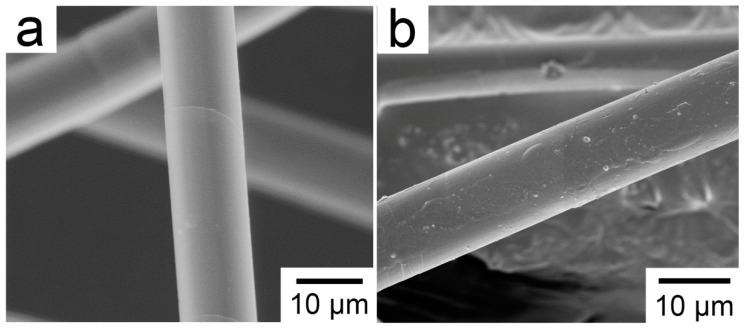
SEM photographs of (**a**) pristine poly(p-phenylene benzobisoxazole) (PBO) fiber; (**b**) pretreated PBO fiber.

Before surface modification, PBO fibers were reflux extraction with ethanol and acetone for 12 h, respectively, dried in a vacuum oven, and then soaked in 60 wt % methanesulfonic acid for one hour at 60 °C. Subsequently, the PBO fibers mentioned above were rinsed with a large amount of distilled water until the pH of the filter liquor was approximately 7; then, they were dried in a vacuum oven for later use. These PBO fibers with similar properties to the pristine ones were simply designated as ‘pretreated PBO fibers’. The SEM photographs of pristine and pretreated PBO fibers are shown in Figure 1. As it can be seen from the comparison of Figure 1a,b, the surface of the PBO fiber is hardly destroyed during the pretreatment. RES (RE solution) modification and coupling agent modification were adopted in this work, respectively. The main components of RES are LaCl_3_ and Ethylene Diamine Tetraacetic Acid (salt). The value of La content was 0.6% in mass fraction. The pretreated PBO fibers mentioned above were immersed in the RES for one hour, and then dried in a 120 °C vacuum oven for 5 h. For the modification of the coupling agent, the pretreated PBO fibers were dipped in the APTES/alcoholic solution with 2% APTES mass fraction at 20 °C for 1 h, then poured out and dried, and finally put it in a 120 °C vacuum oven (DZF-6090) (Shanghai Hecheng Instrument Manufacturing Co., Ltd., Shanghai, China) for 4 h, so that the coupling agent sufficiently formed a complete polysilane layer on the surface of the PBO fiber. The pretreated fibers were applied to blank tests.

### 2.3. Preparation Procedure

The composite sheets were prepared by hot molding. PBO fibers (20 vol %) and PI (80 vol %) were mixed and stirred uniformly. After it was dried under vacuum, the mixture was placed in a metal mold to apply a pressure of 2.1 MPa, a molding temperature of 320 °C, and a holding pressure of 120 min; then, it was demolded to room temperature.

### 2.4. Testing Procedure

The surface of the PBO fiber treated with pretreatment, coupling agent, and RES was then analyzed by -Fourier transform infrared spectroscopy (FT-IR) (Varian Medical Systems, Palo Alto, CA, USA). The difference between the functional groups of PBO fiber before and after surface treatment was compared. The FT-IR scan range was 4000–400 cm^−1^, and the resolution used for the spectra was 0.1 cm^−1^. The surface chemical composition of PBO fiber was characterized by Thermo Scientific ESCALAB250Xi X-ray photoelectron spectroscopy (XPS) (Thermo Fisher Scientific Inc., Waltham, MA, USA). The monochromatic Al K alpha X-ray source (hυ = 1486.6 eV) was used as the XPS excitation source, and the electron binding energy of C1s was 284.8 eV as the internal standard, and the constant analyzer energy (CAE) was 100 eV.

The bending tests adopted the short beam three-point bending test method. The relevant test standard was the Chinese standard GB/T 9341-2000, and the tests were carried out on the INSTRON 3367 Universal Material Testing Machine (INSTRON Corporation, Boston, MA, USA). The test machine head was continuously loaded at a speed of two mm/min until the sample broke. There were more than five effective samples for each composite, and the average value was used as the final result.

The tensile tests were also conducted on a testing machine INSTRON 3367 by adding continuous loading according to the Chinese standard GB/T1040.2—2006. The speed of the testing machine crosshead was one mm/min until the sample broke at 20 °C and 210 °C, respectively. Five samples were tested for each composite, and the effective average was taken.

**Figure 2:**
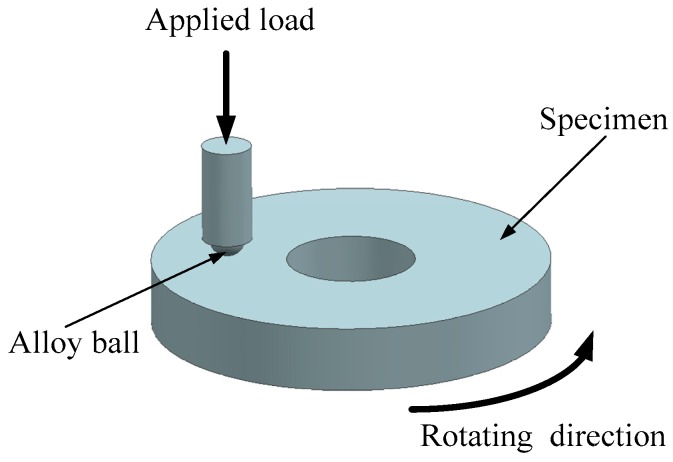
The schematic diagram of the friction pair.

The friction and wear properties of the polyimide polymer matrix composites reinforced by PBO fibers (PBO/PI) were evaluated on a High-Temperature Atmosphere Tribometer (QG-700) with the contact pattern of rubbing pairs of ball–disc contact at 20 °C and 210 °C, respectively. The sample was continuously rotated with the chassis to perform sliding friction with the non-rotating alloy ball, as shown in Figure 2. The counterpart was a hard alloy steel ball with a diameter of 6 mm, a surface roughness of Ra 0.1 μm, and a surface hardness of HRA (a code indicating the hardness of a material) ≥89. The test time was 60 min with the normal load of 6 N, and the sliding speed of 0.5 m/s. The friction reduction performance was represented by the friction coefficient, which was automatically recorded by the tribometer. The wear performance was characterized by the specific wear rate, which was enumerated by the equation K = V/LF, where V was the wear volume (mm^3^) measured by a three-dimensional shape analyzer (NanoFocus μscan, Oberhausen, Germany), F was the normal load (N), and L was the total sliding distance (m). The morphologies of the fracture surfaces and worn surfaces of PBO/PI composites were studied with environmental scanning electron microscope (Philips XL-30, Royal Philips, Amsterdam, The Netherlands).

## 3. Results and Discussion

### 3.1. FT-IR and XPS Analysis of the PBO Fiber Surfaces

**Figure 3:**
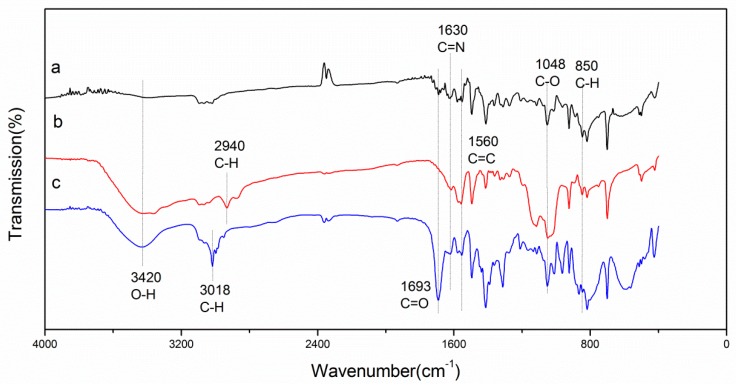
Fourier transform infrared spectroscopy (FT-IR) spectra of the PBO fibers: (**a**) Pretreated; (**b**) Coupling agent; (**c**) Rare earth solution (RES).

Figure 3 displays the FT-IR spectra of the pretreated PBO fiber, coupling agent-treated PBO fiber, and RES-treated PBO fiber. The spectrum has four absorption peaks near 1630 cm^−1^, 1560 cm^−1^, 1048 cm^−1^, and 850 cm^−1^, whether modified or not. The peaks at 1048 cm^−1^ and 1630 cm^−1^ can be ascribed to the stretching vibration of C–O and C=N in the oxazole ring. The peak at 1560 cm^−1^ can be ascribed to the stretching vibration of C=C of the benzene ring. The peak at 850 cm^−1^ can be ascribed to the plane flexural vibration of C–H [24].

In the pretreated PBO fiber spectrum (Figure 3a), a weak peak near 3420 cm^−1^ and 1693 cm^−1^ can be observed, which is due to the O–H and C=O stretching vibrations of the carboxylic acid group, respectively [25]. It reveals that there is a small amount of carboxylic acid group on the surface of the PBO fiber. For PBO fiber treated by coupling agent (Figure 3b), the peak near 2940 cm^−1^ can be ascribed to the stretching vibration of the C–H groups in APTES [26]. The broad absorption peak at 3420 cm^−1^ can be assigned to the stretching vibration of O–H [27], which indicates that there are more hydroxyl groups introduced onto the surface of the PBO fiber via the coupling agent treatment. For the RES-treated PBO fiber (Figure 3c), the peak at 3018 cm^−1^ can be ascribed to the stretching vibration of C–H groups in EDTA [25]. The higher peaks observed near 3420 cm^−1^ and 1693 cm^−1^ are ascribed to the O–H and C=O stretching vibrations of the carboxylic acid group, respectively. This result verifies that a large amount of the carboxylic acid group has successfully grafted onto the surface of the PBO fiber by RES treatment.

**Figure 4:**
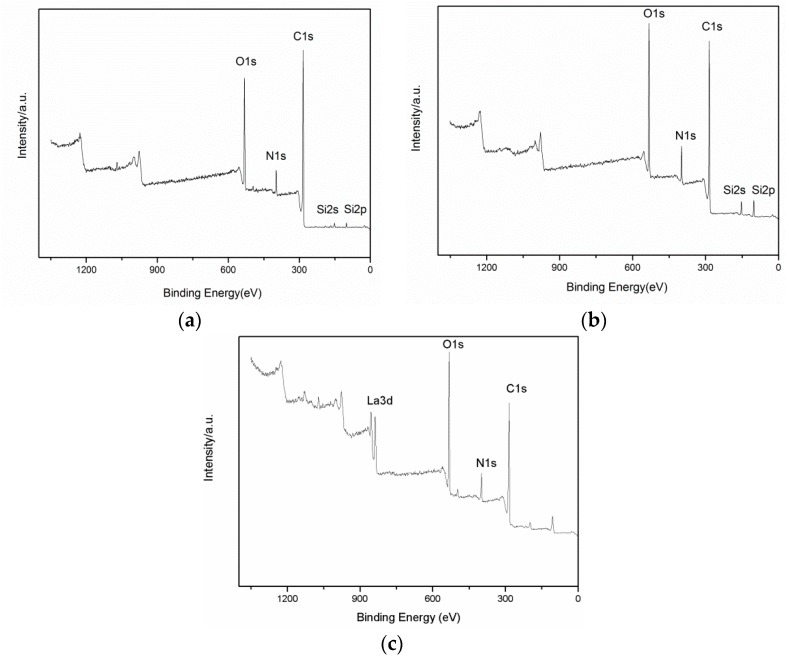
X-ray photoelectron spectroscopy (XPS) spectra of the PBO fibers: (**a**) Pretreated; (**b**) Coupling agent; (**c**) RES.

Figure 4a–c show the XPS spectra of pretreated, coupling agent-treated, and RES-treated PBO fibers, respectively. It can be observed in Figure 4a that the pretreated PBO fiber surface mainly contains C, O, and N elements, as well as a very small amount of Si element remaining after reflux extraction. After the surface treatment of PBO fiber, the intensities of the C1s, O1s, and N1s peaks are changed, which indicates that surface modification changes the elemental composition of the PBO fiber surface. In addition, the increases in the intensities of the Si2p and Si2s peaks in Figure 4b indicate that APTES has covered the surface of the PBO fibers. The appearance of an La3d peak in Figure 4c confirms that La element has covered the surface of the PBO fibers, and the Si2p and Si2s peaks are inconspicuous due to the coverage of the La element.

**Figure 5:**
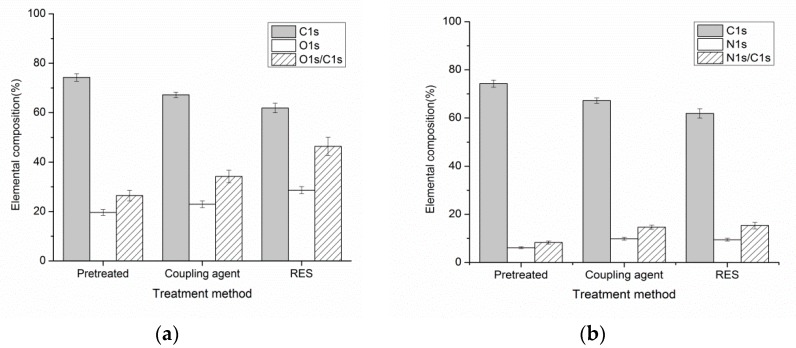
Surface elementary composition of PBO fiber with and without treatment. (**a**) O/C; (**b**) N/C.

Figure 5 represents surface elementary composition of the PBO fiber with and without treatment. As seen from Figure 5, the pretreated PBO fiber surface contains a high concentration of carbon atoms and small amounts of O and N atoms. The atomic ratios of O/C and N/C on the PBO fiber surface all increase after the surface modification. Moreover, RES-treated PBO fiber has the highest value. These results reveal that RES treatment can significantly improve the surface polarity of the PBO fiber, which enhances the physical adsorption ability and the interfacial adhesion between the PI matrix and the PBO fibers.

**Figure 6:**
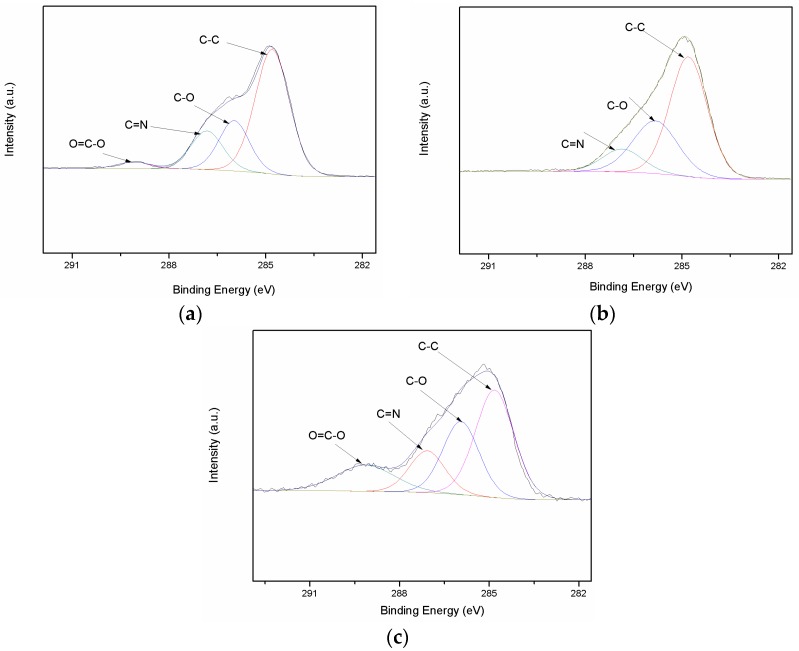
Curve fit of C1s photoelectron peak of the PBO fibers: (**a**) Pretreated; (**b**) Coupling agent; (**c**) RES.

The curve fit C1s spectrum of pretreated, coupling agent-treated, and RES-treated PBO fibers are shown in Figure 6. As shown in Figure 6a, the C1s peak region of pretreated PBO fiber can be deconvoluted into three main fitting peaks with binding energy at 284.83 eV, 285.98 eV, and 286.83 eV, corresponding to the C–C, C–O and C=N groups [22], respectively. A weak peak centered at a binding energy of 289.13 eV is assigned to the O=C–O group. After the surface modification of the coupling agent, the C–O peak area increases obviously, as shown in Figure 6b. For RES-treated PBO fiber, the O=C–O peak area increases significantly compared with that of the pretreated PBO fiber, as shown in Figure 6c. It indicates that RES modification increases the quantity of the carboxylic acid groups on the PBO fiber surface substantially. The relative percentage of the functional groups obtained from C1s peak curve-fitting for pretreated, coupling agent-treated, and RES-treated PBO fibers are listed in Table 1.

The surface modification mechanism of RES and coupling agent treatment could be explained by the following:

1. Due to the large effective nuclear charge, the La atom has great chemical activity, and is apt to combine with the oxygen-containing functional groups such as hydroxyl and carboxyl groups. The La ions undergo coordination chemical reactions with the oxygen-containing active groups on the surface of the PBO fiber during RES surface treatment. Since the coordination number of La is large and variable, the coordination chemical bonding with the organic reactive functional groups (such as amino groups and carboxyl) in the RES can be continued, and the chemical activity of the PBO surface is enhanced by a method similar to chemical grafting.
2. The coupling agent has a bifunctional group in its chemical structure, and its molecular structure can be represented by the general formula RSiX_3_, where X is a hydroxyl group produced by the hydrolysis of the coupling agent. The hydroxyl group can undergo a dehydration–condensation reaction with the hydroxyl group or carboxyl group on the surface of the PBO fiber. Therefore, more hydroxyl groups are introduced to the surface by coupling agent treatment.

### 3.2. Flexural Properties

**Figure 7:**
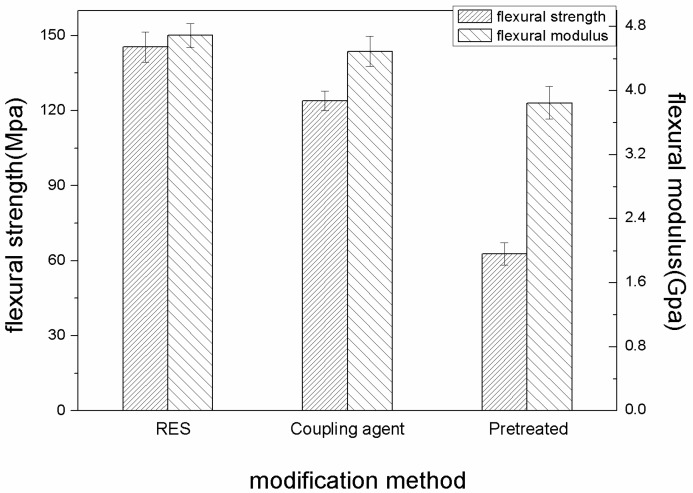
The influence of modification methods on flexural properties at 20 °C.

Figure 7 shows the flexural properties of three kinds of PBO/PI composites at 20 °C.

It is observed from Figure 7 that the surface modification of PBO fibers is an effective method to enhance the flexural strength of the PBO/PI composite. The flexural strength and flexural modulus of the RES-treated composite are improved by about 134.4% and 21.9% compared with that of the pretreated composite respectively, while 99.7% and 16.7% improvements are obtained by treatment with a coupling agent. It reveals that RES treatment can form stronger interfacial bonding between the PBO fiber and the PI matrix, which could transfer load effectively and consume more strain energy before the failure of the PBO/PI composite. RES treatment not only improves the surface activity of the PBO fiber, it also contains other effects as follows. As the coordination reaction of the multiple coordinations of the La element, including the La ions adsorbing to the PBO fiber surface, did not completely occur, they could continue to chemically bond with the oxygen-containing functional groups of the PI molecular chain, such as the carbonyl groups.

**Figure 8:**
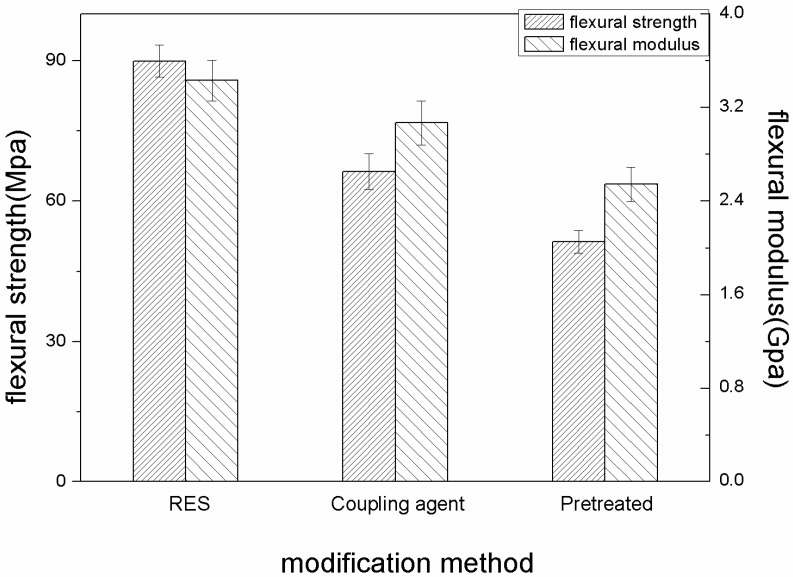
The influence of modification methods on flexural properties at 210 °C.

The flexural properties of three kinds of PBO/PI composites at 210 °C are shown in Figure 8. Obviously, the flexural properties of the PBO/PI composites all decrease compared with that of the composites at 20 °C respectively, and the flexural strength and flexural modulus of the RES-treated composite decreased by about 38.2% and 26.8%, while a 46.4% and 32% decrease were generated by the coupling agent-treated composite. The results reveal that RES treatment is superior to coupling agent treatment in retaining the high-temperature stability of the PBO/PI composite. This could be explained by the following: the electron of the La atom is prone to move between 4f and 6s electronic shells due to the equivalent energy of these two shells, which makes some flexibility exist in the PBO/PI composite interface. The flexible interface plays a role in reducing the stress concentration caused by the elevated temperature.

**Figure 9:**
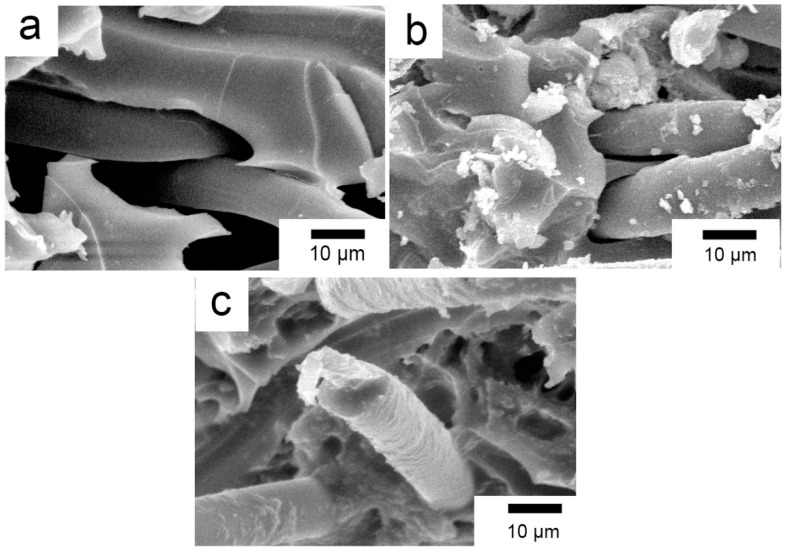
SEM images of the fracture surfaces of composites at 20 °C. (**a**) Pretreated; (**b**) Coupling agent; (**c**) RES.

SEM images of fracture surfaces of the pretreated, coupling agent-treated, and RES-treated composites at 20 °C are shown in Figure 9. For the PI composites reinforced with pretreated PBO fibers, the fiber surface is rather smooth where there is little PI matrix adhered. Meanwhile, it can be observed that the gap between the PBO fibers and the PI matrix on the fractured surface is quite large, as shown in Figure 9a. This suggests that the interfacial adhesion between the PBO fibers and the PI matrix is very poor, and the failure mode of the composite is mainly related to an interfacial taking off adhesive. The interfacial adhesion is improved by coupling agent treatment. It can be observed in Figure 9b that a small amount of PI resin adheres to the surface of the PBO fiber, which is pulled out from the PI matrix. The gap between the PBO fibers and the PI matrix on the fractured surface is decreased compared with that of the pretreated composite. This means that a certain binding force is formed between the PBO fiber and the PI matrix, and the failure mode of the composite is partly changed into the intrinsic failure of the PI matrix. The surface of the RES-treated PBO fiber that was pulled out from the PI matrix become rough, and there is no obvious gap between the PBO fibers and the PI matrix on the fractured surface, as shown in Figure 9c. This reveals that the interfacial adhesion between PBO and PI is further enhanced after RES treatment, so as to exert the reinforcing function of PBO fibers sufficiently. Moreover, it is evident to see that RES treatment is superior to coupling agent treatment in improving the interfacial bond property between the PBO fibers and the PI matrix. As a consequence, the flexural properties of the PBO/PI composite can be promoted considerably.

**Figure 10:**
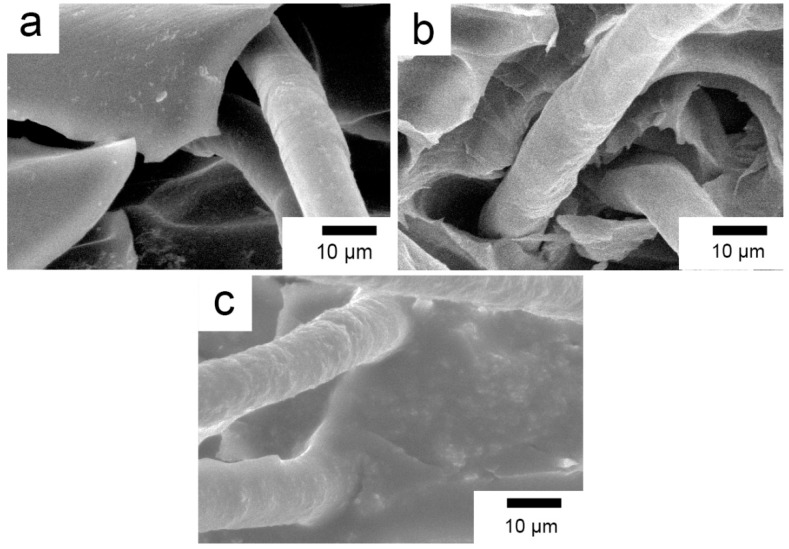
SEM images of the fracture surfaces of composites at 210 °C: (**a**) Pretreated; (**b**) Coupling agent; (**c**) RES.

Figure 10a–c show SEM micrographs of the fracture surfaces of the pretreated, coupling agent-treated, and RES-treated PBO/PI composites at 210 °C, respectively. Compared with the condition of 20 °C (Figure 9a), it can be seen from Figure 10a that the pretreated PBO fiber surface is still smooth with little PI matrix adhered to its surface, and the gap between the PBO fibers and the PI matrix is still very large, which cannot transfer stress from the PI matrix to the PBO fibers effectively. In the case of the coupling agent-treated composite (Figure 10b), the binding performance of the PBO fibers and PI matrix is improved. Compared with the condition of 20 °C (Figure 9b), the gap between the PBO fibers and the PI matrix on the fractured surface is large, which means that the interfacial adhesion between the PBO fibers and the PI matrix is decreased. This result indicates that coupling agent treatment is deficient regarding the composite withstanding high temperature condition. This is the same as Figure 9c; on the fractured surface, the PBO fibers and PI matrix are combined together with no obvious gap between them, as shown in Figure 10c. This suggests that RES-treated PBO fiber has good bonding to the PI matrix at 210 °C, which displays great high-temperature resistance regarding its composite interface. Thus, the PBO/PI composite with optimal high-temperature flexural properties is obtained. The results mentioned above are consistent with the flexural experimental data.

### 3.3. Tensile Properties

**Figure 11:**
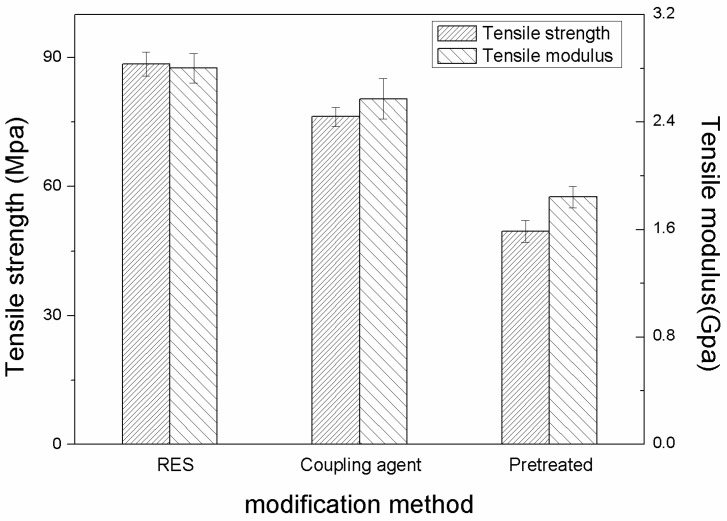
The influence of modification methods on tensile properties at 20 °C.

**Figure 12:**
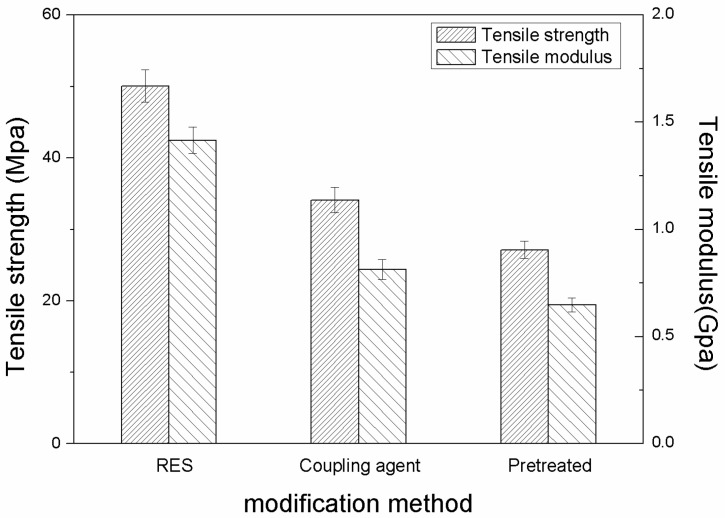
The influence of modification methods on tensile properties at 210 °C.

Figure 11 and Figure 12 display the tensile properties of three kinds of PBO/PI composites at 20 °C and 210 °C, respectively. It can be observed from Figure 11 that the tensile properties of the PBO/PI composites modified by the coupling agent and RES are better than those of the pretreated composite at 20 °C. Compared with the pretreated composites, the tensile strength and modulus of the composite modified by the coupling agent increase by about 53.8% and 39.8%, respectively, while the RES treatment increased the tensile strength and modulus by about 78.6% and 52.1%, respectively.

The tensile properties of all of the PBO/PI composites decreased at different degrees at 210 °C compared with the normal temperature, as shown in Figure 12. However, the tensile properties of the PBO/PI composites modified by the two surface treatment methods were still better than those of the pretreated composite, and the composite modified by RES yielded the best tensile properties. The tensile strength and modulus of the PBO/PI composite modified by the coupling agent were reduced by about 55.3% and 68.4%, respectively, compared with the normal temperature condition (see Figure 11), while the RES treatment reduced the tensile strength and modulus by about 43.5% and 49.5%. The test results show that the two surface treatment methods can improve the interfacial bonding state of the PBO/PI composite, and the stability of the interfacial adhesion obtained by RES treatment is more excellent than that of coupling agent treatment at high temperature.

### 3.4. Friction and Wear Properties

**Figure 13:**
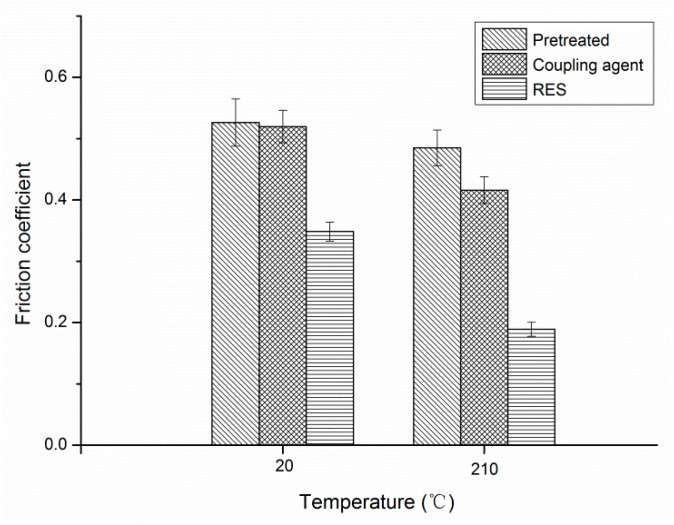
Friction coefficient of composites with different modifications of PBO fibers.

The friction coefficient is mainly related to the adhesion and deformation resistance occurring in the real contact area of the friction pair. When the ambient temperature changes from normal temperature to high temperature, the friction coefficients of the three PBO/PI composites all decrease, as shown in Figure 13. The results of Section 3.2 showed that the increase in temperature caused the mechanical properties of the composite to decrease. The heat accumulated in the contact area caused the friction surface of composite to form an interfacial layer with low shear strength, resulting in a reduction in the friction coefficient. The RES-treated composite had the lowest friction coefficient among the three PBO/PI composites, whether at 20 °C or 210 °C.

**Figure 14:**
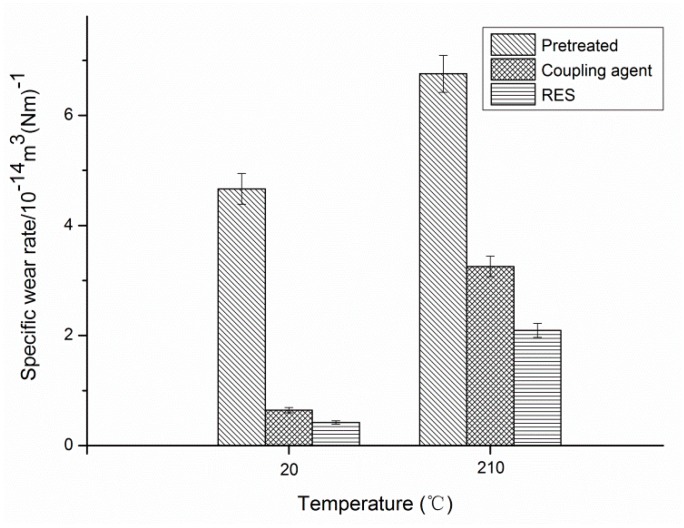
Specific wear rate of composites with different modifications of PBO fibers.

The essence of wear is that the PI macromolecular chain slips or breaks in the action of external force, which then transfers to the counterface or forms free wear debris. It can be observed from Figure 14 that the surface modification of PBO fibers reduces the wear of the composites significantly under normal temperature friction conditions. The high-temperature environment further raises the temperature of the friction surface of the PBO/PI composites that was originally weak in thermal conductivity, which aggravates the adhesion and transfer of the PI resin matrix, resulting in an increase in the specific wear rate of all of the composites. Among them, the specific wear rate of the RES-treated composite reaches the smallest value. This observation indicates that the RES-treated PBO/PI composite still has excellent interfacial adhesion properties in a high-temperature environment, which is beneficial to enhance the wear resistance of the composite.

**Figure 15:**
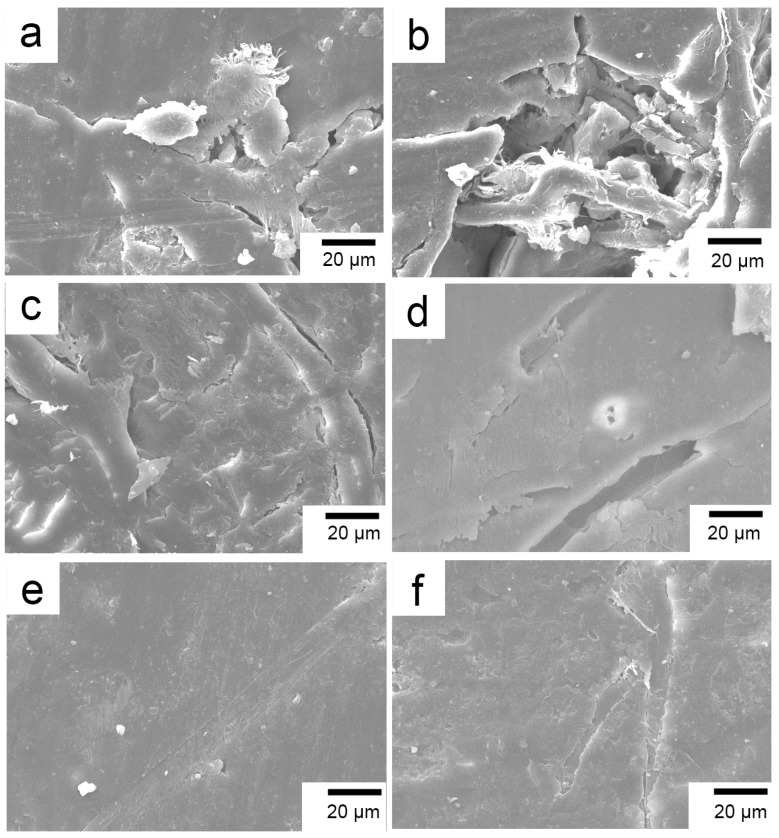
SEM images of the worn surfaces of composites. (**a**) Pretreated, 20 °C; (**b**) Pretreated, 210 °C; (**c**) Coupling agent, 20 °C; (**d**) Coupling agent, 210 °C; (**e**) RES-20 °C;(f) RES-210 °C.

The details of the worn surfaces of composites with different surface modifications of the PBO fibers are shown in Figure 15. For the pretreated PBO/PI composite under normal temperature (Figure 15a), the grooves left on the worn surface due to the detachment of the fibers from the resin matrix indicate the poor interfacial adhesion properties of the composite. The worn surface is mainly characterized by fatigue wear and adhesive wear, and plastic deformation occurs at the same time. The high-temperature environment further exacerbates the adhesive wear of the pretreated PBO/PI composite, and the worn surface has pits left due to the large spalling of the resin matrix, as shown in Figure 15b. The wear degree of the composite reinforced by coupling agent-treated PBO fibers is significantly improved compared with the pretreated composite. Figure 15c shows that wear state under the external load at 20 °C, in which only a small amount of cracks exist between the PBO fibers and the PI matrix, accompanied by the plastic deformation. However, the PBO fibers treated by the coupling agent are pulled out entirely from the resin matrix under the constant action of friction in high-temperature conditions, which indicates that the coupling agent treatment method is not effective at improving the bonding state of the PBO fibers to the PI matrix at a high temperature. Figure 15d–f shows the morphology of the worn surface of the RES-treated composite at ambient temperatures of 20 °C and 210 °C, respectively. It can be seen that the worn surface is smooth under normal temperature conditions; there is no obvious matrix spalling and cracking, and the PBO fibers are tightly combined with the PI matrix, which can effectively bear the load from the friction pair. The wear mechanism is mainly a slight abrasive wear. Under high-temperature conditions, the PBO fibers and the PI matrix can still maintain a good interface bond. The exposed PBO fibers on the friction surface are able to effectively withstand the load from the friction pair. The worn surface is relatively flat and smooth, and mainly characterized by slight fatigue wear and plastic deformation.

## 4. Conclusions

In this study, the effects of RES and coupling agent treatment on the flexural properties and friction and wear behaviors of PI composites reinforced by PBO fibers are investigated under normal and elevated temperature. The results demonstrate that the quantity of the reactive functional groups of the surface-treated PBO fibers was larger than that of the pretreated fibers, and the RES-treated PBO fiber showed the highest reactive functional groups. Accordingly, the RES-treated composite had the best interfacial bonding properties among the three kinds of PBO/PI composites. Moreover, RES treatment is superior to coupling agent treatment in retaining the high-temperature stability of the PBO/PI composites. An excellent interface bond enables the PBO fibers of the friction surface to support the load from the counterpart effectively, thereby reducing plastic deformation and adhesive wear in different extent under normal temperature and high temperature conditions, respectively.

## Figures and Tables

**Table 1:** Relative percentage of functional groups obtained from C1s peak curve fitting of PBO fibers.

Surface Treatment	Relative Percentage of Functional Groups Component (%)				Abbe Criterion
	C–C	C–O	C=N	O=C–O	
pretreated	58.61	21.11	17.27	3.01	0.09
coupling agent	57.51	30.66	11.83	-	0.38
RES	42.23	27.18	15.17	15.42	0.16
